# Polyproline: A Synthetic Polypeptide With Unique Properties

**DOI:** 10.1002/anie.5624080

**Published:** 2026-09-02

**Authors:** Rosanna Le Scouarnec, Josué M. Galindo, Colin Bonduelle, Andreas Heise

**Affiliations:** ^1^ CNRS Bordeaux INP LCPO UMR 5629 University Bordeaux Pessac France; ^2^ Department of Chemistry RCSI University of Medcine and Health Sciences Dublin 2 Ireland

**Keywords:** functional nanomaterials, LCST polymers, NCA polymerization, polypeptides, polyproline

## Abstract

Polyproline is a unique polypeptide defined by its unusual conformational rigidity and physicochemical behavior. Addressing long‐standing challenges in its synthesis and characterization, this mini‐review highlights recent advances in the ring‐opening polymerization of proline *N*‐carboxyanhydride (NCA), enabling the controlled and scalable preparation of well‐defined polyproline architectures. These developments have unlocked diverse applications in advanced materials, including systems that exploit intrinsic thermoresponsive behavior arising from conformational transitions, leading to Lower Critical Solution Temperature (LCST)‐type phase separation with pronounced hysteresis. Emerging applications span smart 3D printed hydrogels, nanoparticles, membrane‐active materials, and polyproline‐based conjugates, where polyproline serves as a biocompatible alternative to poly(ethylene glycol) (PEG). Collectively, these advances position polyproline as a promising platform in biomaterials, offering benefits such as enhanced stability, reduced immunogenicity, and expanded functional versatility.

## Graphical abstract

Poly‐*L*‐proline is a unique polypeptide distinguished by its well‐defined secondary structures and unusual thermal behavior. This mini review highlights recent advances in the synthesis of poly‐*L*‐proline by *N*‐carboxyanhydride (NCA) polymerization, the formation of structurally defined architectures and nanostructures, and emerging strategies that exploit its LCST‐like thermal responsiveness for the development of advanced responsive materials.

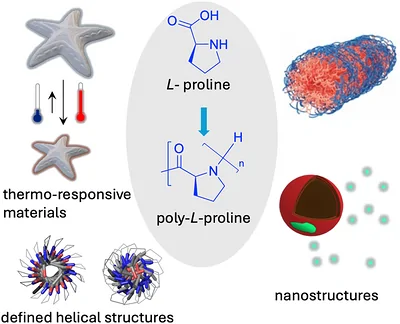

## 1 Introduction

Amino acids are the fundamental building blocks of proteins where they are linked in defined sequences through peptide (amide) bonds. The amino acid sequence or primary structure ultimately determines the three‐dimensional (3D) conformation and biological function of the protein. Through their chemical diversity, amino acids enable proteins to perform essential roles, including enzymatic catalysis, structural support, immune defense, tissue repair, and molecular signaling [1, 2, 3, 4]. From a polymer chemistry perspective, amino acids represent highly attractive monomeric building blocks. Their polymerization yields poly(amino acid)s often referred to as synthetic polypeptides, which represent an original class of polymeric materials interfacing synthetic and natural domains. A variety of monomeric building blocks are available to design polypeptides from the 20 canonical amino acids and more than 200 unnatural ones. Amino acids share structural features such as a carboxylic acid (‐COOH) and an amine group (‐NH_2_) but differ in the nature of their side chain on the central α‐carbon (Cα). The properties of the resulting polypeptides can be tuned depending on the chemical nature of the side chain. For instance, polar or charged side chains can introduce hydrogen bonding and stabilize the polymer chain in aqueous conditions. Hydrophobic residues, on the other hand, avoid interaction with water and introduce weak Van der Waals interactions. In addition to the water‐solubility, the pendant side chain provides a chemically diverse handle for the introduction of specific interactions, post‐polymerization modification, and opportunity to tune many other physical, chemical, and biological properties. Overall, synthetic polypeptides have attracted increasing attention owing to their structures that are analogous to those of proteins. Moreover, they are known for their biocompatibility, biodegradability and versatility compared to other classes of polymers used in the design of nanoparticles, gels or other biomaterials [5, 6].

Polypeptides can be synthesized by two main approaches. Solid‐Phase Peptide Synthesis (SPPS), pioneered by Merrifield in 1963, remains the most widely used technique for the preparation of sequence‐defined peptides [7]. In SPPS, the C‐terminus of the first amino acid is covalently anchored to an insoluble polymeric resin. Chain elongation then proceeds through iterative cycles consisting of N‐protecting group removal, coupling of the next protected amino acid, and washing steps to remove excess reagents and by‐products. SPPS is now highly automated which improved yields and reaction scale [8]. Much better adapted to the preparation of materials, the second approach utilizes a polymerization process called ring‐opening polymerization (ROP) that implies the use of cyclic monomers derived from the amino‐acid precursor, *N*‐carboxyanhydrides (NCA) [9, 10]. The ROP approach is largely motivated by the aim of scalable access (multi gram scale) to functional, biomimetic polypeptides up to high‐molecular‐weights, although at the expense of sequence control. Although a variety of initiators and catalytic systems have been reported [11], primary amine initiation remains the most widely employed method. In this mechanism, the primary amine nucleophilically attacks the carbonyl carbon of the NCA, opening the ring and forming a new amide bond [9]. Subsequent decarboxylation (CO_2_ release) drives chain propagation. Under appropriate conditions, NCA polymerization proceeds in a controlled manner, allowing predictable molecular weight through adjustment of the monomer‐to‐initiator ratio, relatively low dispersity, efficient chain extension, and incorporation of the amine initiator as a defined end group [9, 10]. This synthetic versatility has enabled the preparation of a broad range of polypeptide architectures, including block copolymers [12, 13], star‐shaped polymers [14, 15], macrocyclic backbones [16], surface‐grafted systems [17, 18], nanoparticles [19], and 3D printed structures [20, 21] from many of the proteolytic as well as non‐natural amino acids, thereby expanding the utility of synthetic polypeptides in biomaterials and beyond [22, 23, 24].

*L*‐Proline (Pro) represents a notable exception among all proteogenic amino acids. It is the only amino acid containing a secondary amine, formed by a cyclic aliphatic side chain that covalently links the α‐carbon to the amino nitrogen (Figure 1A). For most amino acids, electrons delocalization through the peptide bond creates a rigid plane with substituents positioned in the energetically favored trans configuration. This delocalization is weaker when proline is involved, and the cis‐configuration has a probability of 5%–6%, which is approximately 100 times higher than for any other amino acid (Figure 1B–D) [25]. As a result, proline‐containing bonds can switch between both conformations, with the cis form found in flexible, surface‐exposed regions of proteins [26, 27]. In addition, the pyrrolidine ring restricts rotation around the N–Cα bond and imposes pronounced conformational rigidity compared to other amino acids [28]. Extended sequences of Pro give rise to two distinct, rigid helical secondary structures: the right‐handed, hydrophobic polyproline I (PP‐I) helix, characterized by cis peptide bonds, and the more prevalent left‐handed, hydrophilic polyproline II (PP‐II) helix (Figure 1C), composed of trans peptide bonds first identified in the 1950s in collagen [29, 30]. The PP‐II helix is particularly abundant in globular, water‐soluble proteins and is often located adjacent to α‐helices or β‐strands [31, 32]. PP‐II conformation is characterized by average backbone dihedral angles of *Φ* ≈ −75° and *Ψ* ≈ +145°. Owing to its highly extended geometry, PP‐II does not typically form the intramolecular hydrogen‐bonding patterns that stabilize classical secondary structures such as α‐helices or β‐sheets. Compared to the compact α‐helix (5.4 Å per turn, 3.6 residues per turn), the PP‐II helix adopts a much more elongated structure, with a helical pitch of approximately 9.3 Å per turn and three residues per turn [33]. While historically named for proline, PP‐II type helical structures also occur in proline‐free peptides and proteins such as collagen [34]. Frequently exposed at protein surfaces, PP‐II segments present their carbonyl and amide groups to the aqueous environment, enabling the formation of regular networks of water‐mediated hydrogen bonds that contribute significantly to stabilizing the overall 3D protein structure [35, 36]. The spontaneous interconversion between cis and trans conformations in natural proteins can result in the compaction/extension of specific parts of the protein [26, 27]. This interconversion can be enzymatically reversed and is involved in the regulation of various physiological and cellular processes [37, 38]. Studies on oligoprolines have shown that the conformational transition between PP‐I and PP‐II are solvent and temperature dependent and occurs via a sequential mechanism involving six intermediate populations [39, 40, 41]. Interestingly, computational analyses have identified two additional secondary structures that are plausible for polyproline: PP‐III and PP‐IV. These two additional helices are based on intermediates between PP‐I and PP‐II, with different torsion angles than those reported for PP‐I and II. PP‐III is a right‐handed helix composed of trans amide bonds, while PP‐IV is a left‐handed helix composed of *cis*‐amide bonds, but so far, they were not isolated experimentally due to their high level of energy [42, 43]. The unique cyclic structure of proline and the conformationally distinct helices of polyproline have long attracted interest from both a fundamental structural perspective and in view of their unusual physicochemical properties as well as for the design of supramolecular materials [44, 45, 46, 47, 48]. Progress in this area was mostly achieved by SPPS synthesis of oligoproline due to the lack of efficient polymerization techniques that enabled reproducible, fast and scalable synthesis of well‐defined high molecular weight polyproline from Pro NCA. Recent synthetic breakthroughs have fundamentally changed this situation, providing reliable access to polyproline with controlled molar mass, architecture, and helicity by Pro NCA polymerization. In this mini‐review, we highlight these synthetic advances and discuss the emerging potential of NCA polymerization‐derived polyproline as an exciting new class of responsive poly(amino acid)s and its potential in polymeric functional materials.

**FIGURE 1:**
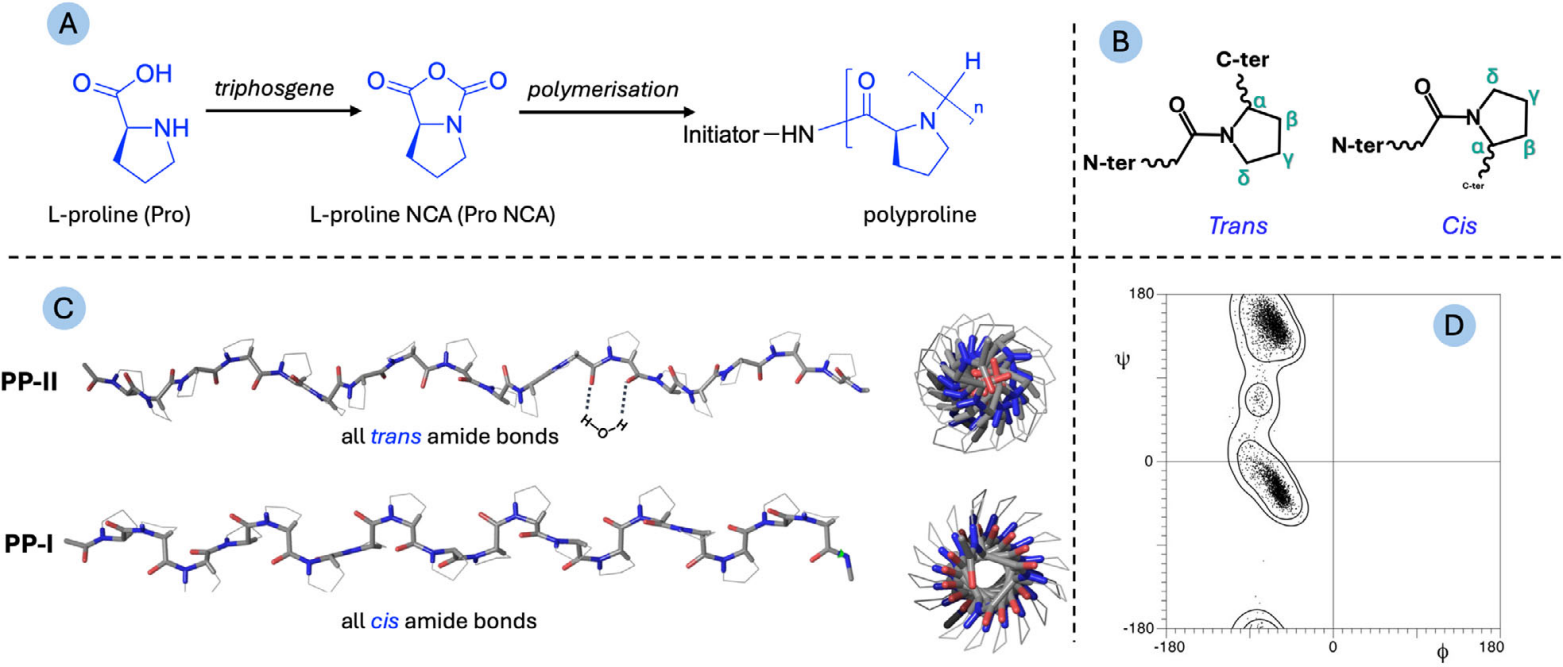
(A) General reaction scheme of the synthesis and polymerization of *L*‐proline *N*‐carboxyanhydride (Pro NCA). (B) Representation of proline trans and cis configuration. (C) Molecular models of the two secondary structures PP‐II and PP‐I, corresponding lateral and axial view of the secondary helices. (D) Ramachandran plot representing probabilities for torsion angles values *Ψ* and *φ* in proline residues. Reproduced from Lovell et al. [28].

## 2 Synthesis of Poly(L‐proline) by NCA Polymerization

Despite the first report on the polymerization of Pro NCA in 1954 [49], this monomer has remained comparatively underexplored, largely due to difficulties associated with its preparation, purification, and controlled polymerization. To date, Pro NCA synthesis using triphosgene remains the most efficient approach despite its toxicity. Early studies demonstrated access to both low‐ and high‐molar‐mass polyproline through Pro NCA polymerization, however, reactions often required up to 1 week, provided poor control over molar mass, and involved laborious work‐up procedures [50, 51]. The outcome of Pro NCA polymerization is highly sensitive to reaction parameters, including solvent polarity, monomer concentration, temperature, and initiator type. These factors strongly influence chain solubility, propagation kinetics, and the onset of premature precipitation potentially arising from the formation of the PP‐I helix, frequently resulting in loss of control over the process. Subsequent methodological advances have addressed some of these limitations. These include the use of transition‐metal catalysts to enhance control [52], a method that requires an inert atmosphere and is usually carried out in a glove box. A significant improvement was also obtained by synthetic routes to highly pure Pro NCA via *N*‐*tert*‐butyloxycarbonyl (*N*‐Boc)‐protected *L*‐proline [53, 54]. Impurities such as nucleophiles or bases negatively impact NCA stability and in particular the sensitive Pro NCA. Therefore, highly pure Pro NCA not only improved storage stability but also enabled the preparation of well‐defined polyproline homo‐ and block copolymers with improved molar‐mass control [55, 56]. More recently, Lu and coworkers reported a water‐assisted, controlled polymerization of Pro NCA in a binary acetonitrile (ACN)/water system, affording well‐defined PP‐II helices at a 100 mg scale [57]. In this medium, polymerization proceeds rapidly within minutes, and poly(*L*‐proline) remains soluble throughout the reaction, facilitating access to well‐defined polymers (Figure 2A,B). The authors demonstrated the robustness of the system by grafting polyproline for various amine‐bearing proteins (Figure 2C). However, control over molecular weight could not be achieved for degrees of polymerization > 200 [47]. Mechanistic studies revealed that water plays an active role by facilitating proton transfer during chain propagation, thereby lowering the activation barrier and significantly accelerating the polymerization process. Importantly, the polymerization proceeds in a controlled manner, enabling precise tuning of the polyproline molecular weight and facilitating subsequent chain extension to access more complex architectures. To date, a broad range of primary amine initiators has been successfully employed following this approach, including small‐molecule initiators, multifunctional cores for the preparation of star‐like structures, and biomacromolecules such as proteins, thereby highlighting the versatility of this approach for advanced macromolecular and bioconjugate design. This approach provides convenient and reliable open vessel route to polyproline architectures and is increasingly adopted for the synthesis of polyproline. A drawback of the aqueous binary solvent system is the competing hydrolysis of the NCA monomer, which can affect molecular weight control for slower reacting systems or when higher molecular weights are targeted. The method is therefore most effective for fast polymerizing systems as often observed for multifunctional initiators. Very recently the Lu group reported that stoichiometric addition of acetic acid acts as a multifunctional stabilizer in Pro‐NCA monomer in dichloromethane [58]. While the polymerization is slower as compared to the ACN/water system, excellent polymerization control was achieved for homo as well as copolymerizations. In this system, the acetic acid solubilizes and stabilizes the growing PP‐II helix via hydrogen bonding, catalyses the ring‐opening polymerization of Pro NCA with high in situ selectivity of trans amide, and enhances chain end fidelity by improving the selective addition of carbonyl.

**FIGURE 2:**
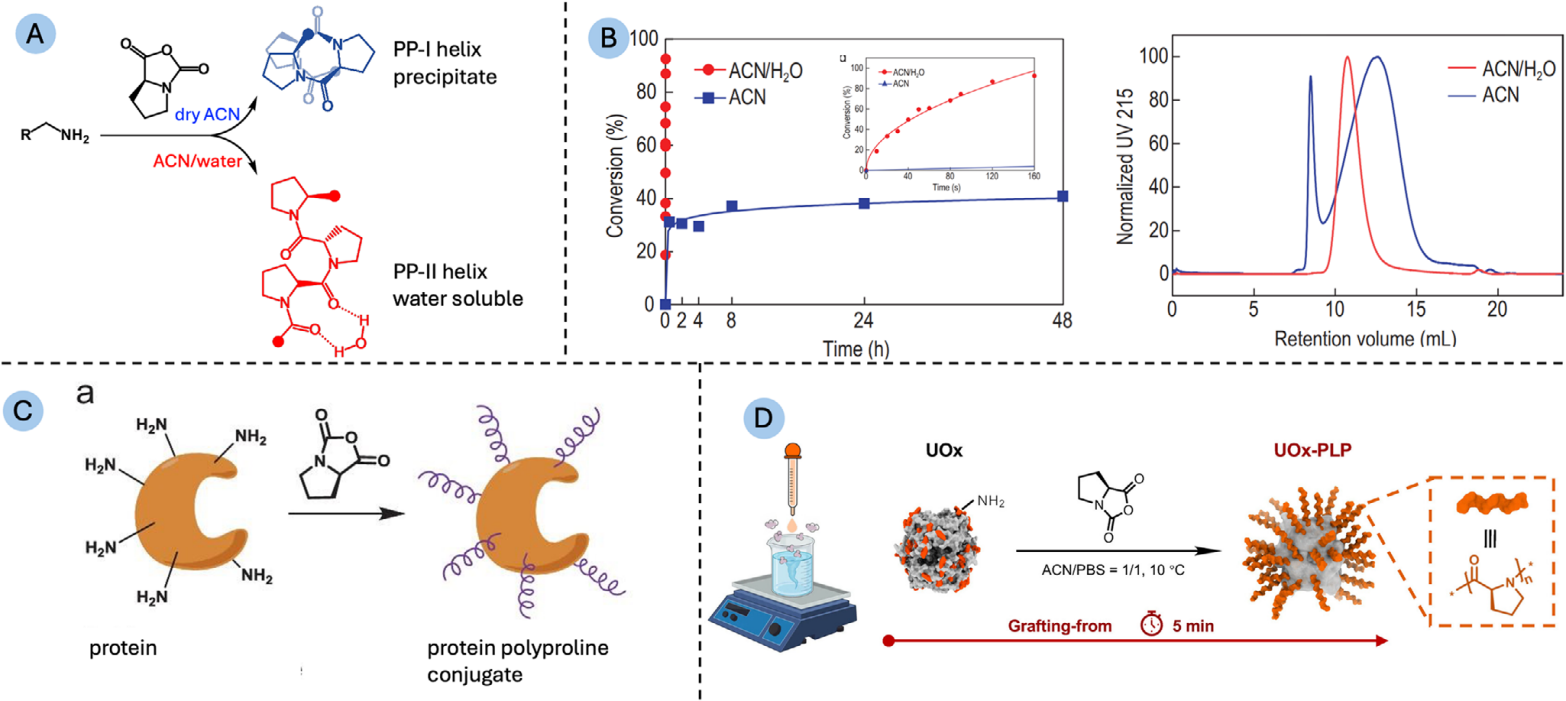
(A) Comparison of ROP of Pro‐NCA in pure dry ACN and ACN/water and geometric representations of resulting PP‐I and PP‐II helices. Water interaction with PPII is represented on two adjacent carbonyls. (B) Pro NCA conversion over time and zoomed‐in period, showing accelerated NCA consumption in case of ACN/water (left). Adapted from Hu et al. [57]. Size Exclusion Chromatography (SEC) of produced polyproline, showing biomodal traces when synthesized in ACN, and narrow monomodal traces in case of ACN/water. (C) Scheme of protein‐initiated ROP of ProNCA in ACN/water. Adapted from Hu et al. [57]. (D) Synthetic scheme of UOx‐PLP using the grafting‐from approach. Adapted from Hu et al. [65].

Although synthetic advances have significantly expanded the structural diversity and architectural complexity of polyproline‐based materials, their characterization remains challenging. The pronounced chain rigidity of polyproline severely limits its solubility in common organic solvents. In particular, the compact PP‐I helix is essentially insoluble in most media, whereas the more extended and relatively hydrophilic PP‐II conformation can only be dissolved in aqueous environments or selected water–organic solvent mixtures, and often only to a limited extent. Such non‐standard solvent systems are not universally accessible, for example for the molecular weight determination by size exclusion chromatography (SEC), which frequently precludes reliable analysis by this technique. Moreover, the intrinsic backbone rigidity and restricted conformational mobility adversely affect spectroscopic analysis. In solution‐state NMR spectroscopy, reduced chain mobility leads to signal broadening and limited resolution, sometimes complicating structural elucidation and, in particular, end‐group analysis required for determining the degree of polymerization. Experimentally, most techniques are suitable for oligomers and low molecular weight polymers, while the assessment of high molecular weight polyproline is challenging. Spectroscopy techniques, such as circular dichroism (CD), FTIR or NMR are frequently applied [30, 54, 55, 59, 60, 61, 62, 63]. In the context of CD, the PP‐II structure is characterized by a strong negative band at 205 nm and a weak positive peak around 228 nm. In contrast, PP‐I exhibits three distinctive bands: a negative one around 200 nm, a strong positive peak at 213 nm and a weak negative band at 232 nm [30]. FTIR can detect specifically the amide I and II bands, respectively corresponding to C═O stretching and N─H bending/C─N stretching [64]. In the PP‐I structure, amide I and II bonds have characteristics bands at 1620 and 1428 cm^−1^, respectively. In the PP‐II structure, they are reported at 1641 and 1433 cm^−1^ [54]. ^1^H, ^13^C, or N─H NMR have also been used to study the cis/trans conformation of proline oligomers in solution and is certainly the best tool to shed light on the dynamics of the isomerization [59, 61, 62, 63].

## 3 Advanced Materials From Poly(*L*‐proline)

### 3.1 Materials Inspired by Polyproline in Proteins

The initial interest in polyproline centered on elucidating its distinctive helical conformations and associated physicochemical properties, from which potential applications were subsequently derived. Owing to the pronounced rigidity of the PP‐II helix in aqueous solution, polyproline was proposed as a molecular ruler for structural biology [66]. However, detailed spectroscopic investigations revealed conformational heterogeneity, calling into question its suitability for the precise determination of absolute intermolecular distances [67, 68]. Further inspiration emerged from the role of proline‐rich motifs in natural proteins. Notably, antifreeze glycoproteins found in extremophilic organisms adopt secondary structures resembling the PP‐II helix [69, 70]. Ice recrystallization inhibition (IRI) activity is of considerable technological importance, with applications ranging from the cryopreservation of cells and tissues to large‐scale uses such as aircraft de‐icing. Weak IRI activity of low‐molecular‐weight polyproline was first reported by Gibson and coworkers [71]. Building on advances in the efficient polymerization of Pro NCA in ACN/water mixtures, the same group investigated a series of polyprolines with theoretical molar masses ranging from 2000 to 50 000 g mol^−^^1^ [72]. The highest‐molar‐mass samples inhibited ice growth at significantly lower concentrations than previously studied proline oligomers, which failed to completely suppress recrystallization even at elevated concentrations. This enhanced activity has been attributed to a combination of factors, including stabilization of the PP‐II helical conformation, polymer aggregation, and selective binding to specific ice crystal faces. Collectively, these findings demonstrate that polyproline represents an effective, scalable, and potentially more sustainable alternative to protein‐based ice recrystallization inhibitors. More data will be needed to assess how practical and economical polyproline as an antifreeze additive is.

A second example inspired by the role of polyproline motifs in natural proteins arises from their stabilizing function at the protein‐water interface. Heise, Bonduelle, and coworkers postulated that polyproline could similarly stabilize polypeptide nanostructures in aqueous media [73, 74]. To realize this concept, they employed ring‐opening polymerization‐Induced Self‐Assembly (ROPISA), whereby chain extension of a solvophilic macroinitiator, typically in water, with a soluble monomer generates an amphiphilic block copolymer that spontaneously self‐assembles into nanostructures with defined morphologies such as vesicle and worm‐like structures [75, 76, 77]. While PISA is well established in controlled radical polymerization, its combination with NCA polymerization for the preparation of polypeptide nanostructures has only recently been demonstrated [74, 78, 79]. Using NCA‐based ROPISA, the first synthetic polyproline‐stabilized polypeptide nanoparticles were prepared via rapid polymerization of Pro NCA in ACN/water employing multifunctional eight‐arm initiators, followed by chain extension with benzyl‐*L*‐glutamate (BLG) NCA or Z‐*L*‐lysine (ZLL) NCA to induce self‐assembly (Figure 3A). This one‐pot strategy enabled the efficient synthesis of well‐defined star‐shaped polyproline‐based block copolypeptides within minutes, affording anisotropic worm‐like and spherical nanoparticles with sizes of approximately 60 nm [73]. Importantly, the resulting nanostructures exhibited compartmentalized secondary structures: the nanoparticle core adopted either a poly(γ‐benzyl‐*L*‐glutamate) (PBLG) α‐helical or poly(Z‐*L*‐lysine) (polyZLL) β‐sheet conformation, whereas the polyproline corona maintained a PP‐II helix. These nanoparticles were further shown to encapsulate both hydrophobic and hydrophilic dyes, highlighting their potential as versatile peptidomimetic platform materials for nanomedicine applications. They represent an advantage over other sytems due to their fast and open vessel sytntheis as well as the fact that they are fully derived from amino acids. This ROPISA approach was also extended by Bonduelle and coworkers in pure aqueous media (Figure 3B) [74]. Without prior chemical modification, and using simple homopolymers of proline, aqueous ROPISA was achieved with BLG‐NCA to afford thermoresponsive nanomaterials exhibiting anisotropic shapes. Interestingly, the thermal property of polyproline is preserved in this approach, allowing the aggregation of nanoparticles to be controlled simply by changing the temperature, with a chracteristic hysteresis.

**FIGURE 3:**
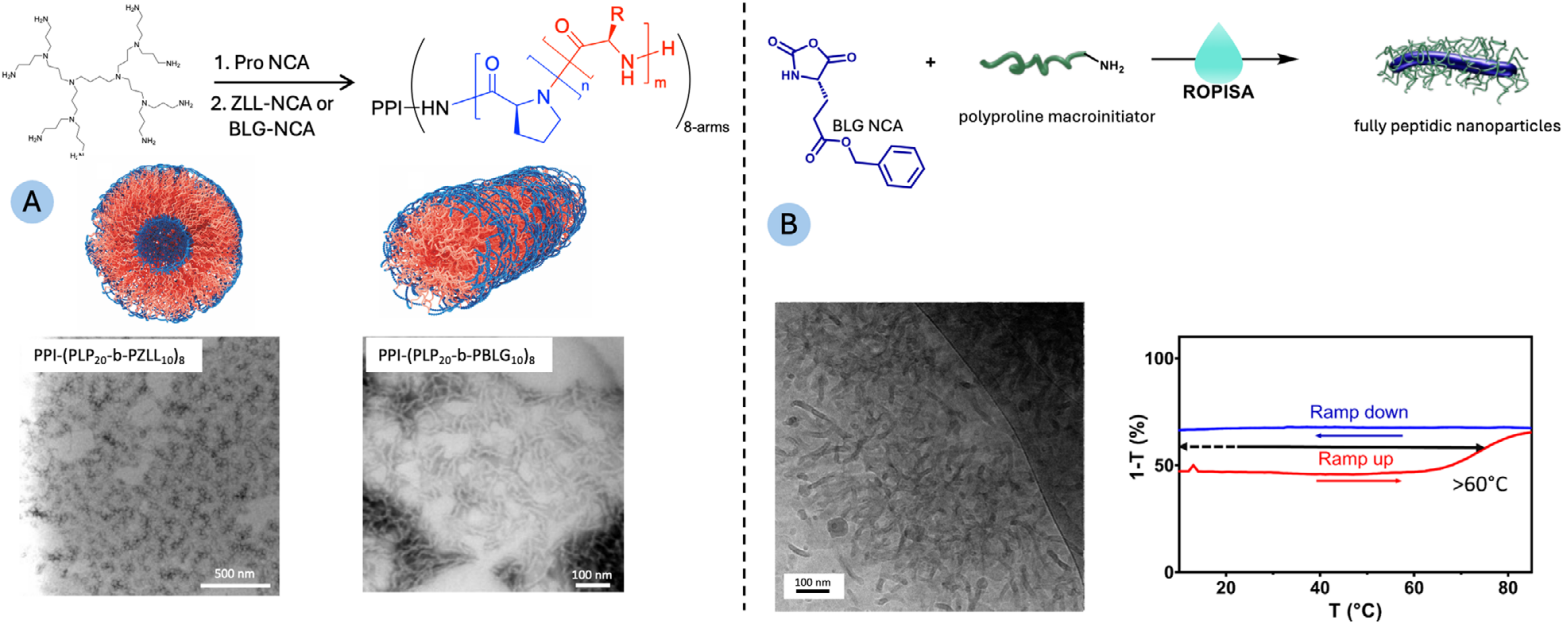
Polyproline nanomaterials by amphiphilic diblock polypeptides synthesized in one‐step aqueous ring‐opening polymerization‐induced self‐assembly (ROPISA). (A) Multiarm block copolymers of Pro NCA and benzyl‐*L*‐glutamte or Z‐*L*‐lysine NCA in ACN/water (1:1, v/v). Transmission electron microscopy (TEM) images of nanoparticles and proposed nanostructures. Adapted from Tinajero‐Diaz et al. [73]. (B) Nanomaterials obtained by amphiphilic diblock polypeptides self‐assembly. Cryo‐TEM image of worm‐like structure nanomaterials and transmission‐normalized (*λ*_abs_ = 550 nm) turbidimetry curves for PLP‐*b*‐PBLG solution as function of temperature in pure water at 0.4 mg mL^−1^. Adapted from Beauseroy et al. [74].

A similarly stabilizing role of polyproline in protein therapeutics was more recently reported by Lu and coworkers, who explored its use to improve pharmacokinetics and safety profiles [65]. Conventionally, such improvements are achieved through conjugation with poly(ethylene glycol) (PEG) in a process known as PEGylation [80]. PEGylation involves the covalent attachment of PEG chains to bioactive molecules, including proteins, peptides, and small‐molecule drugs. This modification alters the physicochemical and biological properties of the parent compound, typically enhancing aqueous solubility and colloidal stability. In vivo, PEGylation can prolong systemic circulation time while shielding the biomolecule from proteolytic degradation and immune recognition, features that are particularly advantageous for protein therapeutics. Despite its longstanding status as a gold standard, PEGylation has come under increasing scrutiny due to PEG's non‐biodegradability and its tendency to elicit anti‐PEG antibodies, which may lead to accelerated blood clearance (ABC) [81, 82]. The growing prevalence of anti‐PEG antibodies raises potential clinical concerns, prompting the search for alternative hydrophilic polymers [83]. Among the candidates investigated, polypeptides have attracted attention as PEG substitutes in protein conjugation [84, 85, 86, 87]. They offer intrinsic biodegradability, structural biomimicry, and precise structural tunability and side‐chain functionality. Notably, Lu and coworkers observed that conjugation of a therapeutic protein with a helical polypeptide significantly improved pharmacological performance, including enhanced binding affinity, stability, and in vivo efficacy. Moreover, these helical polypeptide conjugates elicited a markedly reduced immune response and exhibited a substantially weaker ABC effect compared to both non‐helical polypeptide counterparts and PEG‐modified analogues [88]. Building on these findings, the group subsequently explored polyproline as a naturally occurring, helical, and hydrophilic polypeptide for protein conjugation. Employing a grafting‐from strategy, solvent‐exposed lysine residues on the enzyme uricase were used to initiate the ROP of proline NCA in an ACN/water solvent mixture, yielding uricase densely functionalized with polyproline chains (Figure 2D). Uricase was selected due to its clinical relevance in the treatment of hyperuricemia and gout, disorders associated with the pathological accumulation of uric acid. The enzyme catalyses the oxidation of poorly soluble uric acid to the more soluble and readily excreted allantoin. However, it is not naturally present in humans, which contributes to its immunogenicity. Using the grafting‐from approach, approximately ten polyproline chains with molecular weights of up to ∼5000 g/mol were introduced per uricase molecule. While surface grafted polyproline form a physical barrier that blocks proteases and immune receptors they still allowed the small substrates to diffuse into the catalytic pocket retaining ∼82% of the native enzymatic activity. The resulting polyproline corona markedly enhanced thermal stability, resistance to lyophilization and freeze–thaw stress, and protection against proteolytic digestion compared with both the unmodified enzyme and PEGylated versions. In vivo, in direct comparison with a PEGylated analogue, the polyproline conjugate induced minimal anti‐drug antibody formation and exhibited no accelerated blood clearance (ABC) in rats, even upon repeated administration. Its pharmacokinetic profile and uric acid–lowering efficacy remained unchanged in animals previously sensitized to the PEGylated counterpart. In a hyperuricemia model, the polyproline‐modified enzyme maintained therapeutic efficacy over multiple doses, whereas the PEGylated control showed a progressive decline in activity. Overall, the rapid synthesis, enhanced stability, and excellent nonclinical performance highlight the translational potential of urinase–polyproline conjugates and demonstrate the broader promise of the grafting‐from strategy for the development of improved therapeutic enzymes.

### 3.2 Materials Utilizing the Thermal Properties of Polyproline

Macromolecules, whether natural or synthetic, can undergo phase separation in solution when a thermodynamically stable, homogeneous system transitions into an insoluble state. Among the various triggers for phase separation, temperature plays a central role, particularly for polymers exhibiting a lower critical solution temperature (LCST) [89]. These polymers are soluble in water below the LCST. Upon heating above this temperature, dehydration of the polymer chains occurs, leading to aggregation and macroscopic phase separation. As a result, two immiscible phases coexist in thermodynamic equilibrium [90]. The temperature at which phase separation becomes optically detectable is commonly referred to as the cloud point temperature (*T*_CP_). In contrast, the LCST is a thermodynamic parameter that defines the temperature below which the polymer remains soluble at all concentrations. Prominent examples of synthetic LCST polymers include poly(*N*‐isopropylacrylamide) (PNIPAM) and poly(2‐oxazoline)s, both of which have been extensively investigated for responsive materials applications [91, 92]. In biological systems, increasing temperature typically results in protein denaturation and irreversible aggregation. Notable exceptions are certain intrinsically disordered proteins, such as elastin‐like polypeptides (ELPs), which display reversible temperature‐induced phase separation [93]. For synthetic polypeptides derived from natural amino acids, LCST behavior is rarely observed. Instead, thermoresponsiveness is typically introduced through side‐chain functionalization with LCST‐active moieties, such as oligo(ethylene glycol) units or tertiary amines, thereby imparting temperature‐dependent solubility [94]. For a more comprehensive discussion of thermoreponsive polymers with focus on proteins and NCA‐derived synthetic polypeptides the reader is referred to a recent review article [94].

Polyproline is unique among polypeptides in exhibiting an intrinsic LCST‐type phase behavior without the need for side‐chain modification. This phenomenon was first reported in the 1960s by Ciferri and Orofino, who observed that oligoprolines adopting the PP‐II conformation precipitated from aqueous solution at elevated temperatures [95]. Although resolubilization was not systematically investigated at that time, subsequent studies noted that redissolution occurred at temperatures substantially lower than the apparent LCST. This behavior is highly unusual, as conventional LCST systems typically exhibit phase separation and redissolution at, or very close to, the same temperature. The first systematic investigation of the LCST behavior of polyproline was later conducted by Bonduelle and coworkers using high‐molar‐mass linear polyproline (up to 20 000 g mol^−^^1^) [56]. They reported a distinctive and unprecedented thermoresponsive profile in aqueous solution, with a cloud point (*T*_CP_) at 65°C–70°C upon heating, while resolubilization occurred at a temperature approximately 50°C lower, defining a separate clearing temperature (T_CL_) (Figure 4A). This pronounced thermal hysteresis was remarkably robust with respect to polymer concentration and chain length. In contrast to classical LCST polymers such as poly(*N*‐isopropylacrylamide), where phase separation is driven primarily by enthalpy‐entropy changes associated with polymer‐water interactions, the LCST behavior of polyproline is typically attributed to a temperature‐induced conformational transition. Specifically, heating triggers conversion from the hydrophilic PP‐II helix (all‐trans amide bonds) to the more hydrophobic PP‐I helix (cis amide bonds), resulting in aggregation (Figure 4B). Spectroscopic analyses provided further insight into the observed hysteresis behavior. FTIR measurements revealed a trans‐to‐cis amide bond transition at the cloud point, while circular dichroism indicated an increased fraction of PP‐I helical segments. Notably, a mixed PP‐I/PP‐II population persisted during cooling until the clearing temperature was reached, at which point the hydrophilic PP‐II conformation again dominated and the polymer redissolved. The pronounced hysteresis therefore originates from the conformational transition between PP‐I and PP‐II helices. This was further corroborated by the fact that copolymers derived from racemic D/*L*‐proline, which did not adopt a defined secondary structure and showed no thermoresponsive behavior. This mechanism is unprecedented among LCST materials and imparts a distinct “memory effect” to polyproline‐based systems. More recently it was also reported that changes of the medium with incorporation of salts can be used to tune PLP transition temperatures [96].

**FIGURE 4:**
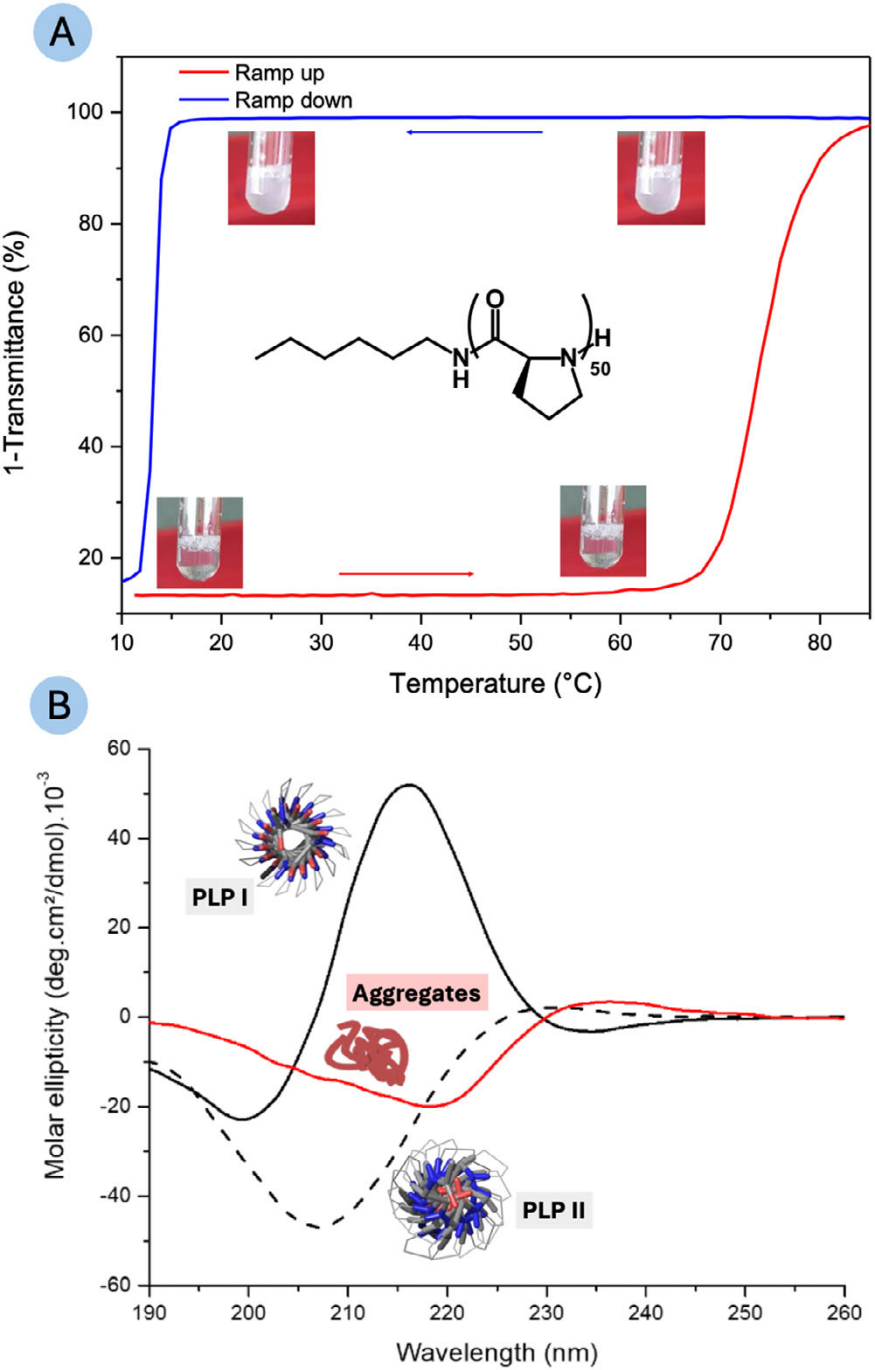
(A) PLP_50_ UV–vis turbidimetry curves as function of temperature, and corresponding images of PLP solution during thermal heating/cooling process (red/blue curves, respectively). (B) CD spectra in water of PPI (black), PP‐II (dotted line) and their modelized structure (axial views), and aggregates (red). Adapted from Badreldin et al. [56].

Particularly this LCST property paves the way to design smart polypeptide‐based materials and researcher have begun to exploit that. One area of interest is thermoresponsive hydrogels. Olsen and coworkers investigated physical hydrogels from a concentrated solution of polyproline in water [55]. They observed self‐supporting hydrogels for polymer concentrations of 10% and above. The solutions were reported to gel at low temperatures, within the range −15°C to 5°C and upon heating under physiological temperatures accompanied by a change in gel stiffness.

In more recent work, thermoresponsive hydrogels based on star‐shaped polyproline were reported by Murphy and coworkers and successfully processed via digital light processing (DLP) 3D printing (Figure 5) [97]. This work represents the first examples in which the intrinsic, conformation‐driven LCST behavior of polyproline was translated into a structurally defined, photocrosslinked network suitable for additive manufacturing. The authors synthesized well‐defined four‐arm polyproline star polymers by controlled ROP of proline NCA. Subsequent end‐group functionalization with methacryloyl moieties afforded photocrosslinkable macromonomers compatible with DLP printing. This strategy enabled the fabrication of hydrogel constructs with high shape fidelity and tunable network architectures. Importantly, the molar mass of the polyproline arms directly influenced both printability and thermoresponsive behavior, highlighting the close interplay between macromolecular design and processability. The resulting printed hydrogels retained the characteristic LCST‐type response of polyproline in aqueous media. Upon heating, a conformational transition associated with PP‐II to PP‐I helix conversion induced changes in hydration and network structure, leading to reversible volume and mechanical alterations. Similar to the linear polyproline the thermoresponsive behavior here is directly coupled to secondary structure transitions. Notably, the printed objects exhibited temperature‐triggered shape morphing, demonstrating that the conformational dynamics of polyproline can be harnessed in macroscopic constructs. This study establishes polyproline as a viable building block for advanced, stimuli‐responsive hydrogel materials and its integration into additive manufacturing platforms thereby expanding the scope of polyproline‐based smart materials beyond solution‐phase studies toward functional, structurally defined soft devices.

**FIGURE 5:**
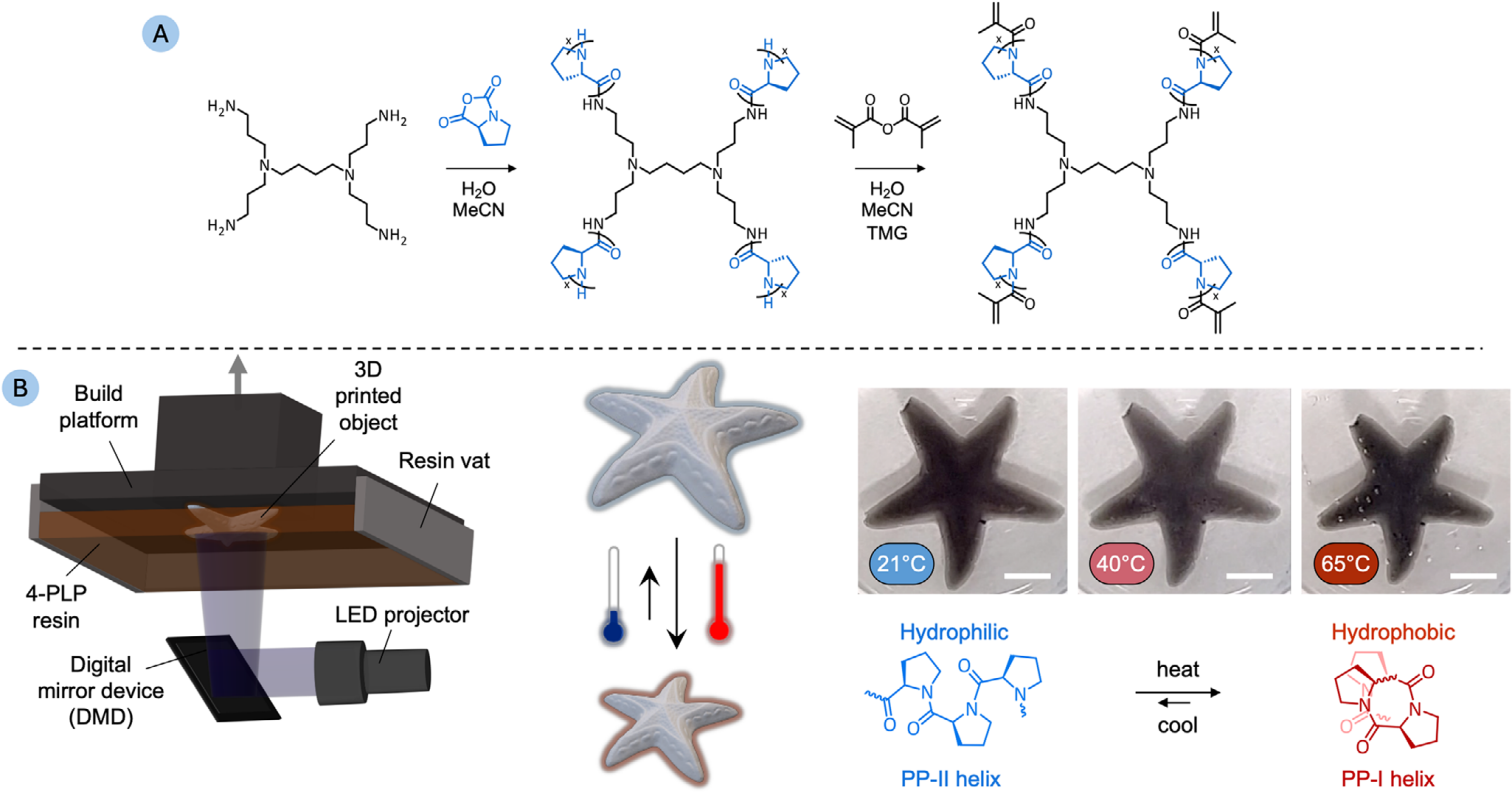
(A) Synthetic route for generation of four‐armed star polyproline crosslinkers suitable for 3D Digital Light Processing (DLP). (B) Graphic of DLP printer, whereby 4‐arm polyproline aqueous resins were printed into target 3D start fish structures. Thermal shrinkage and hysteresis of 3D starfish, and images of thermoresponsive shape morphing of DLP printed starfish (scale bar: 3.0 mm) by reversible PP‐II to PP‐I secondary structure transition. Adapted from Murphy et al. [97].

In a general approach, conjugation between lipids and small peptides or proteins, mostly through peptidic coupling or electrostatic interactions, have been studied for therapeutics approaches and the preparation of liposome nanocarriers [98]. In a biomimicry approach, lipoproteins can be envisaged as polypeptide backbones displaying one or several lipid functions either at the terminal extremity of the chains or decorating the pendant residues of the amino acids along the polypeptide backbone. Their synthetic analogues, called lipopolypeptides can be synthesized through various synthetic pathways like NCA ROP [11]. The main advantage of ROP in this process is that lipid functionalization can be introduced in a ‘one‐pot’ approach when used as the polymerization initiator. Several examples have been reported using lipophilic amines initiating Z‐lysine‐NCA [99, 100], or BLG‐NCA [101] ROP. Optimizing ROP conditions can also be tedious to ensure proper kinetics and control over ROP. For instance, Barz and coworkers reported the improved ROP conditions for lipid‐polysarcosine conjugates synthesized in benzonitrile, using bisalkylated C_14_ and C_18_ amines as initiators [102]. This synthetic approach relying on ROP has also been used as a prebiotic model to investigate and better understand the N‐acylation of proteins. To that end, Izgu and coworkers studied the polymerization of valine‐NCA in presence of fatty acids such as octanoic acid and oleic acid in a buffered mixture where oleic acid forms bilayer [103]. Here, lipid functionalization is used as an anchor for membrane insertion of lipopolypeptides. Other studies have used such strategy to alter membrane properties [104]. Given the growing interests in designing protein‐like polymers to better tune membrane permeability [105] and the chemical advantages in using polypeptide‐based materials to do so, a polyproline backbone is highly interesting to finely control membrane permeability. Indeed, in Nature proline‐rich sequences are involved in cell‐penetrating peptide design [106]. Bonduelle and coworkers explored the design of lipopolyproline conjugates through Lu's adapted aqueous ROP of Pro NCA (Figure 6A). The synthetic procedure was efficiently improved by adding a base to the initiator, ensuring its proper nucleophilicity, and lipopolyprolines (Lipo‐PLP) were characterized using DOSY NMR and MALDI‐TOF techniques to confirm the phospholipid end chain. Resulting lipopolymers demonstrated typical polyproline hysteretic LCST behavior above a critical DP of 50 [56, 107]. Transition temperatures, especially the cloud point, was sharply decreased to 45°C by hydrophobic end chain tethered to the PLP backbone, a trend previously reported with other thermoresponsive polymers [56, 94, 108]. In comparison, *T*_CL_ value was preserved, suggesting no or unsignificant influence of lipid end chain. Fluorescent Lipo‐polyprolines were also prepared and embedded through electroformation in phospholipid bilayers or polymer giant vesicles membrane at various concentrations [107]. Imaging revealed that the lipopolymers colocalized with membrane staining regardless of its concentration, suggesting its homogeneous distribution within the bilayers. The group also demonstrated that such membrane‐anchored polyproline induces microdomain formation within vesicles’ membrane when heated at 60°C, above *T*_CP_ (Figure 6B). The heating process led to an overall change in lipo‐polyproline fluorescence localization, which was then distinguishable from membrane staining, indicating a clear phase‐separation between phospholipids and lipo‐PLP. This protein‐like behavior was quite robust, as the polymer aggregates remain stable at room temperature during all confocal microscopy observations. Noteworthy, a cooling process below the T_CL_ restored Lipo‐polyproline and membrane staining colocalization throughout the membrane, showing the disappearance of the phase separation and the recovery of homogenous vesicles as initially prepared. This result indicated the thermal reversibility of the aggregation process (Figure 6B). These findings were also correlated to lipo‐polyproline lateral diffusion within the membrane and showed that by modulating critical transition temperatures *T*_CP_ and *T*_CL_ to induce membrane reorganization, it is possible to trigger and mimic polyproline hysteretic LCST on the surface of vesicles. Concomitantly, microdomain formation was associated with a selective diffusion of hydrophilic compounds inward and outward (Figure 6C), including macromolecules such as polymers and enzymes [107]. This study highlights Lipo‐PLP potential as lipoprotein mimics for local control of membrane permeability, which could have a positive impact on the development of synthetic cells. However, the current system requires a temperature above the biological temperature to control overall membrane permeability. While the group also demonstrated the robustness of the system in nanoscaled liposomes as potential advanced systems for drug delivery [109], further structural optimization will be required to match it to a biologically relevant temperature range, for example mild hyperthermia, that is, 39°C–42°C [110].

**FIGURE 6:**
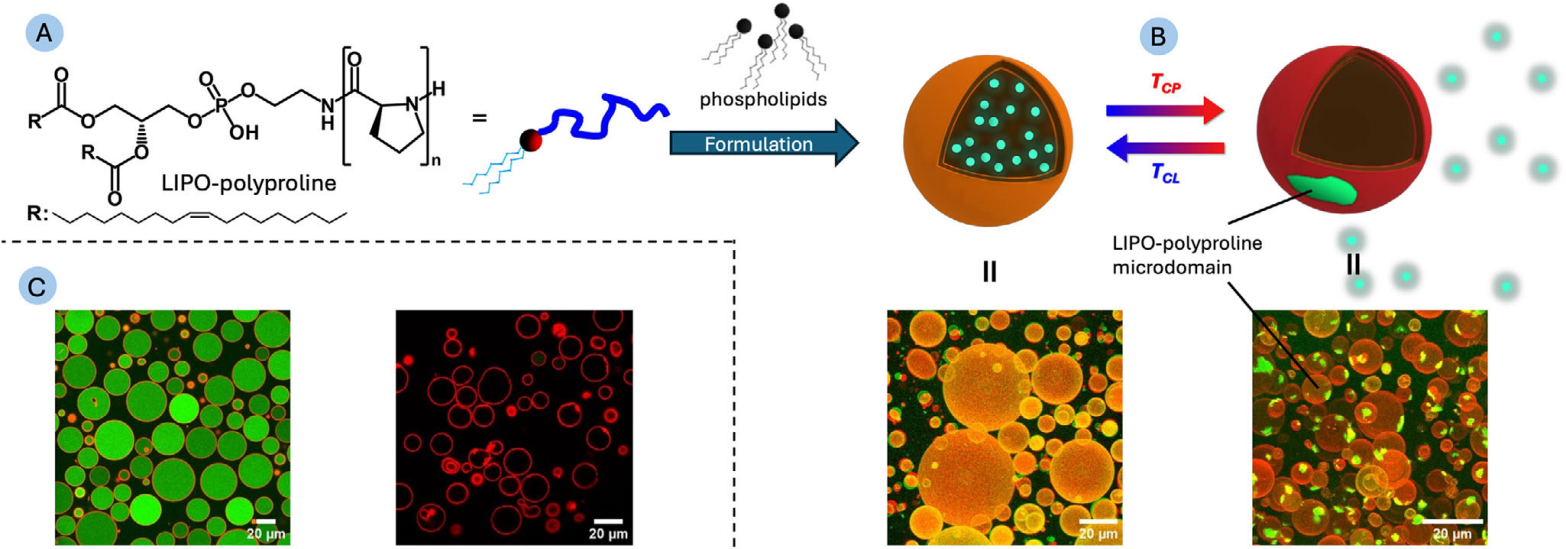
(A) Lipo‐polyproline structure and formulation of thermoresponsive Giant Unilamellar vesicles. (B) Stacks of vesicles at room temperature after electroformation (left) and after heating (right). Lipo‐polyproline was labelled with FITC to visualize microdomain formation upon heating and Lipo‐PLP is redispersed upon cooling (image not included), scale bar = 20 µm. (C) Confocal images of vesicles encapsulating calcein before (left) and after (right) heating, demonstrating calcein release after heating step. Scale bar = 20 µm. Adapted with permission from Journal of the American Chemical Society, 2025, 147, 24213 [107]. Copyright 2025 American Chemical Society.

## 4 Functional Polyproline From NCA Polymerization

Collagen, the most abundant protein in animals, relies heavily on 4‐hydroxyproline (Hyp), a functional proline derivative, for its structure and function. Hyp is an amino acid that stabilizes PP‐II helices and promotes the formation of the collagen triple helix [111, 112]. Previous studies have synthesized and examined protected forms of polyHyp. However, existing methods were limited to relatively low molecular weights and linear structures [113, 114]. Very recently, different approaches have enabled the synthesis of high molar mass PHyp through adapted ROP [52, 58, 115], including protection‐free synthesis of non‐linear polyHyp. In all these strategies, stabilizing the growing PP‐II helix via H‐bonding was key to efficiently synthesizing functional polyproline analogues [58]. Other side‐chain functionalization could be also useful for modulating intrinsic polyproline properties. In this regard, the design and synthesis of clickable proline monomer units were accomplished using either azide [116] or alkyne [96, 117] moieties to generate a polyproline platform that allows post‐polymerization functionalization or conjugation. These included triethyleneoxy‐grafted polyprolines, which were found to be soluble in a wide range of solvents (both organic and aqueous). Importantly, such chemical modifications can also influence macromolecular properties. Conversion of azide groups to triazoles has been shown to modulate solubility, intermolecular interactions, and thermal responsiveness. In certain cases, these modifications stabilize the PP‐II helix or modulate stimuli‐responsive behavior, including lower critical solution temperature (LCST)‐type transitions [118]. The synthesis of functional proline derivatives, their conversion to NCAs, and their controlled polymerization are extremely challenging. It remains still unclear if these approaches will gain broader acceptance in the synthetic polypeptide community. However, clickable proline comonomers could be valuable for designing advanced polyproline materials, motivating further investigation and optimization of their synthesis.

## 5 Conclusions and Outlook

Polyproline is a unique polypeptide, featuring conformational flexibility that gives rise to specific physicochemical properties. Historically, Pro‐NCA polymerization was considered one of the most challenging NCA polymerizations because the growing polymer rapidly adopts ordered helical conformations and precipitates. By addressing this issue, recent advances in controlled ROP techniques have significantly improved access to well‐defined polyproline architectures, enabling systematic exploration of their behavior. Recent research on polyproline has focused primarily on understanding its unusual conformational behavior, solvent‐dependent helicity, and the mechanistic aspects of proline NCA ring‐opening polymerization. This research has particularly examined the role of water‐assisted and controlled polymerization pathways. Meanwhile, application‐driven research leverages the rigid PP‐II helical structure of polyproline to develop biomaterials, collagen‐mimetic scaffolds, drug delivery systems, and functional peptide nanostructures with tunable self‐assembly and biocompatibility. These recent developments have revealed a broad range of applications, from thermoresponsive materials and hydrogels to nanostructures and biomimetic systems. Specifically, the intrinsic LCST behavior and conformational switching of polyproline present new opportunities for designing smart, stimuli‐responsive materials. However, translating these materials into real applications will require a deeper understanding of the relationships between structure and properties, as well as the long‐term stability, bioactivity, and scalable manufacturing of polyproline‐based biomaterials. Overall, its biocompatibility and protein‐like characteristics position polyproline as a promising alternative to conventional polymers in therapeutic and biomedical applications. We anticipate that ongoing advancements in synthesis, characterization, and functional integration will further unlock the potential of polyproline in the fields of advanced material science and biotechnology.

## Author Contributions

**Rosanna Le Scouarnec**: writing – review and editing, resources. **Josué M. Galindo**: writing – review and editing. **Colin Bonduelle**: writing – original draft, visualization, supervision, conceptualization. **Andreas Heise**: writing – original draft, visualization, supervision, conceptualization.

## Conflicts of Interest

The authors declare no conflicts of interest.

## Use of Generative AI and AI‐Assisted Technologies in the Writing Process

During the preparation of this work, the authors used ChatGPT in order to improve readability and language. After using this tool, the authors reviewed and edited the content as needed and take full responsibility for the content of the publication.

## Data Availability

Data sharing not applicable to this article as no datasets were generated or analyzed during the current study.
